# Psychological Distress, Loneliness, and Boredom Among the General Population of Tyrol, Austria During the COVID-19 Pandemic

**DOI:** 10.3389/fpsyt.2021.691896

**Published:** 2021-06-10

**Authors:** Franziska Tutzer, Beatrice Frajo-Apor, Silvia Pardeller, Barbara Plattner, Anna Chernova, Christian Haring, Bernhard Holzner, Georg Kemmler, Josef Marksteiner, Carl Miller, Martin Schmidt, Barbara Sperner-Unterweger, Alex Hofer

**Affiliations:** ^1^Division of Psychiatry I, Department of Psychiatry, Psychotherapy and Psychosomatics, Medical University Innsbruck, Innsbruck, Austria; ^2^Department of Psychiatry, Central Hospital, Sanitary Agency of South Tyrol, Bolzano, Italy; ^3^Department of Psychiatry and Psychotherapy B, State Hospital Hall in Tyrol, Hall in Tyrol, Austria; ^4^Department of Psychiatry and Psychotherapy A, State Hospital Hall in Tyrol, Hall in Tyrol, Austria; ^5^Department of Psychiatry, County Hospital Kufstein, Kufstein, Austria; ^6^Department of Psychiatry, County Hospital Lienz, Lienz, Austria; ^7^Division of Psychiatry II, Department of Psychiatry, Psychotherapy and Psychosomatics, Medical University Innsbruck, Innsbruck, Austria

**Keywords:** COVID-19, psychological distress, pandemic, loneliness, boredom

## Abstract

**Background:** COVID-19-related mental health problems are considered a public health challenge. The aim of this study was to investigate psychological distress, loneliness, and boredom among the general population of the federal state of Tyrol, Austria.

**Methods:** Residents of Tyrol aged ≥ 18 years were recruited via dissemination of a link through social media and other advertisements and invited to complete an online survey from June 26th to August 20th, 2020. Next to the collection of sociodemographic and COVID-19 related variables the Brief Symptom Checklist (BSCL), the Three-Item Loneliness Scale (TILS), and the Multidimensional State Boredom Scale-Short Form (MSBS-SF) were used to assess psychological distress, loneliness, and boredom.

**Results:** 961 participants took part in the survey (68.3% woman). Of these, 14.4% were burdened from psychological distress (BSCL), 22.6% reached a TILS score ≥ 7 and were therefore classified as severely lonely, and boredom levels lay by a mean of 25.9 ± 11.0 points in the MSBS-SF (range: 7–56). Women, singles, low-income people as well as those who were unemployed were significantly more often affected by all of the selected outcomes compared to the remaining sample and they had significantly more frequently consumed alcohol or other substances since the outbreak of the pandemic in order to feel better. In addition, young and middle-aged adults were particularly burdened by loneliness and boredom.

**Discussion:** Our findings identify vulnerable groups and factors associated with higher psychological distress, loneliness, and boredom in the context of the pandemic. In order to prevent mental health problems it will be critical to identify options of maintaining social contacts and remaining active despite pandemic-related restrictions.

## Background

COVID-19 reached Austria and in particular the federal state of Tyrol in February 2020, when an Italian couple living in Innsbruck was tested positive for SARS-CoV-2 after returning from Lombardy, a region in northern Italy that has been one of the most affected areas at the beginning of the pandemic (1). Next to general recommendations like the mandatory use of protective mouth/nose masks in public places, distance keeping, and vigilant hand washing, the Austrian government meanwhile has imposed three lockdowns (March 16th–April 7th, November 3rd–December 6th, December 26th–January 24th) that were associated with a number of confinements, e.g., travel restrictions, cancellation of events, school and university closure, restaurant closure, etc. as well as quarantine and exit restrictions. Obviously, such measures result in profound changes in people's everyday life like disruption of daily routines and those affected may experience a lack of personal freedom and develop unhealthy lifestyle behaviors (2).

Notably, the term “stress” is not understood or felt in the same way in all cultures (3). It is a dynamic process between body and mind when the requirements of a situation are greater than the available resources. Accordingly, individual cognitions and environmental evaluations play a central role in this process (4). Following Lazarus and Folkman, the relationship between personal and environmental factors and personal coping strategies also affects the extent to which a situation is perceived as stressful. Thus, the extent to which a stressful situation is perceived as such depends not only on the situation itself, but also on personal beliefs, characteristics and perceptions (5). Furthermore, the experience of new conditions, unpredictability, threat to self, and loss of control are thought to trigger neurophysiological stress responses (6). For the purpose of this study, we therefore assumed that the COVID-19 pandemic can be seen as a global stressor. The study population and all people worldwide face an uncertain future both privately and economically and thus, the pandemic is perceived as a threat to life, whether financially, socially or physically (7).

Quarantine is generally an important tool for disease control, but it is often associated with a negative impact on mental health (8). A number of studies have shown that the psychological distress caused by isolation can last for months or years after quarantine (9, 10) and is frequently associated with increased levels of anxiety, depression, frustration, insecurity, agitation, sleep disturbances, and boredom (8), the latter of which having a reinforcing effect on perceived and emotional stress (11). Accordingly, it is not surprising that boredom has been reported to be of major relevance in the context of a pandemic like SARS and COVID-19 (12, 13).

Boredom is experienced when an activity is perceived as under- or overwhelming or of low value (14). Bored people may experience a loss of control over their environment, may feel separated and therefore be more aware of their psychological problems (15). They show a higher susceptibility to cognitive and affective dysregulation (16–18), depressive symptoms and anxiety (19–21), are more likely to break the rules of social isolation, and have an increased risk to use drugs (11). On the other hand, social isolation per se is a challenge for people who use drugs or alcohol in harmful ways (22) and it can trigger substance use among vulnerable groups of people who have experienced trauma or mental health problems in the past (23).

Next to boredom, disconnection from society and social contacts in the context of quarantine may cause loneliness (24), which represents a further risk factor for mental and physical illness (24, 25). Of note, the mortality rate of lonely adults is comparable to that of obese people and smokers (26) and loneliness has been related to suicidal intentions and parasuicidal behavior (27). However, increasing social connectedness does not necessarily lead to a reduction of loneliness (28).

In the meantime, a number of studies from all over the world have described the impact of the COVID-19 pandemic on mental health (29). A survey from China, for example, reported on moderate to severe depressive or anxiety symptoms in 16.5 and 28.8% of 1,210 study participants, respectively. In addition, more than half of study participants rated the psychological impact of the pandemic as moderate to severe (30). Similarly, an increased prevalence of fear, anxiety, and depression was observed in the United states (31), Chile (32), and various European countries, e.g., France and Italy (33, 34). Pieh et al. investigated a representative sample of the Austrian general population during the first lockdown and found depressive symptoms in 21.0%, anxiety symptoms in 19.0%, and insomnia in 15.7% of 1,005 study participants (35).

The Tyrolean ski resort Ischgl played a critical role in the pan-European spread of the pandemic in March 2020. Because of that, five Tyrolean communities (Ischgl, See, Kappl, Galtür, and St. Anton am Arlberg) were quarantined on March 13th, and the entire federal state of Tyrol (population of 757,634) was quarantined from March 19th to April 7th. In order to expand on the above mentioned findings of Pieh et al. and focusing on the population of Tyrol, the current ongoing longitudinal study aims to investigate the psychological impact of the COVID-19 pandemic and associated quarantine measures over a 12-month period. In addition, we aim to investigate whether these impacts are affected by sociodemographic and individual-level factors and whether modifiable factors (e.g., resilience, social support) moderate the effects of the pandemic over time. Focusing on the associations of sociodemographic and COVD-19 related variables with psychological distress, loneliness, and boredom, we report here the cross-sectional findings obtained in the 8-week baseline assessment. Other data related to resilience, extraversion, spirituality, and emotion regulation strategy usage will be presented in other reports. Future longitudinal data will be collected and will be reported at a later stage.

## Methods

### Study Population

Residents of Tyrol aged ≥ 18 years were recruited via dissemination of a link through social media and other advertisements and invited to complete an online survey from June 26th to August 20th, 2020 (baseline assessment). Up to the end of recruitment, 3,920 SARS-CoV-2 cases (active + recovered) were recorded in Tyrol (36). Electronic data capture was conducted by means of the Computer-based Health Evaluation System (CHES), a web-based software program that enables electronic data assessment in routine practice and clinical trials (37). Data were collected in an anonymized manner and included both sociodemographic data as well as standardized questionnaires. Ethical approval was obtained by the ethics committee of the Medical University Innsbruck. Participants were provided with a written consent before completing the questionnaires and they were asked to provide their email addresses in order to be reminded for follow-up investigations. Provision of email addresses was not a prerequisite to participate in the baseline survey. At the end of the survey, participants received a downloadable information sheet on professional support numbers and addresses.

### Sociodemographic and COVID-19 Related Variables

In the first part of the survey, sociodemographic data were collected, including age, gender, educational level, marital status, urbanicity, work status, annual household income, type of housing, number of people living in the same household, care of minors as well as personal and family history of psychiatric disorders. In addition, some COVID-19 related data were collected, e.g., whether participants had been tested for SARS-CoV-2 and how severe symptoms had been in case of a positive test result. Another pool of data collected regarded the perception and acceptance of containment measures as well as substance use since the outbreak of the pandemic. Lastly, participants were asked whether they felt exposed to violence or whether their propensity for violence had increased during confinement.

### Psychological Distress

Psychological distress was assessed using the 53-item Brief Symptom Checklist (BSCL) (38). The BSCL is a Likert type scale and the items are scored from 0 (not at all) to 4 (extremely). It measures nine symptom patterns of mental health problems (somatization, obsession-compulsion, interpersonal sensitivity, depression, anxiety, hostility, phobic anxiety, paranoid ideation, and psychoticism). The Global Severity Index (GSI) used in the current study was calculated using the sums of the nine symptom dimensions plus four additional items not included in any of the dimension scores divided by the total number of answered items. As recommended by the authors of this instrument, GSI T scores ≥ 63 were considered as clinically relevant psychological distress. The BSCL has shown good to satisfactory internal consistency for all subscales (Cronbach's α ranging from 0.70 to 0.89) and excellent external consistency for the GSI score (α = 0.96) (39).

### Loneliness

Loneliness was measured by using the short form of the Revised University of California Los Angeles (R-UCLA) Loneliness Scale, the Three-Item Loneliness Scale (TILS) (40). It consists of the questions: “How often do you feel that you lack companionship?,” “How often do you feel left out?,” and “How often do you feel isolated from others?.” Responses include “Often,” “Some of the time,” and “Hardly ever or never.” The TILS total score ranges from 3 to 9 with higher scores indicating a higher degree of loneliness (40). Based on previous studies, scores ≥ 7 were defined to indicate severe loneliness whereas a score of 5 or 6 was defined to indicate moderate loneliness (41, 42). The TILS has demonstrated satisfactory internal consistency (Cronbach's α = 0.72) and high concurrent and discriminant validity (40).

### Boredom

Boredom was assessed using the Multidimensional State Boredom Scale-Short Form (MSBS-SF) (43), which consists of eight Likert-type items that are rated on a 7-point scale (1 = strongly disagree, 7 = strongly agree), yielding a maximum score of 56. Higher scores indicate a higher degree of boredom (43). The MSBS-SF has shown excellent internal consistency (Cronbach's α = 0.909) and good discriminant validity (44).

### Statistical Analysis

Data were analyzed using SPSS, version 26. All statistical tests were performed at a 0.05 level of significance (two-tailed). The primary aim of the analysis was to investigate the association of sociodemographic and COVID-19 related variables with psychological distress, loneliness, and boredom. Psychological distress and loneliness were dichotomized for this purpose (GSI T score <63 vs. ≥ 63, TILS score <7 vs. ≥ 7, respectively), whereas the MSBS-SF total score was used for analyzing boredom (without dichotomization).

Comparisons of participants with normal and elevated GSI T and TILS scores with respect to sociodemographic and COVID-19 related variables were conducted using the Chi-square test. Odds ratios (OR) were determined as a measure of effect size. MSBS-SF scores in dependence of sociodemographic and COVID-19 related variables were analyzed by the two-sample *t*-test for variables with two categories and by one-way analysis of variance for variables with three or more categories. Cohen's d was used to quantify effect sizes.

Logistic regression analysis was used to investigate the combined effects of sociodemographic and COVID-19 related variables on psychological distress and loneliness. Only those variables that had reached a *p*-value < 0.1 in the above analyses were considered as independent variables. The stepwise backward elimination method was used for the identification of significant predictors. Similarly, we used linear regression to analyze the combined effects of sociodemographic and COVID-19 related variables on boredom. Only those variables that had reached a *p*-value < 0.1 in the univariate analysis were entered as independent variables. Significant predictors were identified by stepwise backward variable elimination.

To test for a potential common method bias (respondents' views simultaneously affect independent and dependent variables) we performed an explanatory factor analysis (EFA) on the pooled set of COVID-19 related independent variables and the dependent variables (psychological distress, loneliness, and boredom). We then calculated the proportion of the total variance explained by the first factor in the EFA, where usually a value ≥ 50% is considered as an indication of common method bias.

### Power Analysis (GPower 3.1.9)

Of the 961 persons included, ~900 had complete data on important sociodemographic, COVID-19 related, and psychological variables. Under standard conditions regarding type-one error and power (alpha = 0.05, 1-beta = 0.8), this sample size is sufficiently large to detect the following effect sizes. For comparing two groups with regard to a binary outcome variable by Chi-square test, the sample size allows detection of an OR of 2.39, if the proportions of both variables involved stay within the interval. The latter condition was fulfilled for most subgroups considered. For two-group comparisons with regard to metric outcome variables, the sample size permits detection of a Cohen effect size of d = 0.31. For binary logistic regression analyses, the sample size is sufficient to detect an OR of 1.70 under fairly liberal conditions [that the probability *P* (y = 1) for the dependent variable y under the null hypothesis is ≥ 0.05, and that *R*^2^ for all additional covariates is ≤ 0.4]. For linear regression analyses, testing for an increase in *R*^2^ and allowing for up to 10 degrees of freedom (d.f.) for the predictors involved in testing and up to 30 d.f. for the total set of independent variables, the sample size allows detection of an *f* ^2^ of 0.0182. All of these effect sizes are small according to Cohen's classification of effect sizes (45).

## Results

### Sociodemographic and Health-Related Variables

Nine hundred sixty-one members of the general population of Tyrol (68.3% female) with a mean age of ~42 years and a mean education of ~15 years participated in the survey. Table 1 gives an overview of sociodemographic and health-related variables. Forty-seven study participants lived in places with high exposition to COVID-19 like Ischgl or St. Anton am Arlberg. 14.4% suffered from psychological distress (GSI T), 30.9% experienced moderate and 22.6% even severe loneliness (TILS), and they reached a mean of 25.9 ± 11.0 points in the MSBS-SF. The number of people living in the same household did not significantly affect the outcomes.

**Table 1:** Sociodemografic and health-related variables (*N* = 961).

**Variable**	**Mean ± SD or *N* (%)**
**Gender**	
Male	303 (31.6%)
Female	654 (68.3%)
Others	1 (0.1%)
Age (years)	41.9 ± 13.9 (18–96)
Education (years)	15.4 ± 3.7
**Relationship**	
Single	247 (25.8%)
Fixed partnership	710 (74.2%)
**Children**	
None	630 (65.9%)
1	136 (14.2%)
2	149 (15.6%)
≥3	41 (4.3%)
**Work situation**	
Full-time	473 (49.4%)
Part-time	188 (19.6%)
Self-employed	44 (4.6%)
Education/training	55 (5.7%)
From home	13 (1.3%)
Short-time work	23 (2.4%)
Unemployed	10 (1.0%)
Retired	93 (9.7%)
Homemaker	17 (1.8%)
Others	41 (4.3%)
**Household income**	
<25,000 /year	349 (36.3%)
25,000–49,999 /year	359 (37.4%)
≥50,000 /year	220 (22.9%)
Not specified	33 (3.4%)
**Place of residence**	
Urban (Innsbruck, >100,00 inhabitants)	303 (31.5%)
Village or small town	600 (62.4%)
Places with high exposition to COVID-19	47 (4.9%)
Not specified	11 (1.1%)
Flat size (m^2^)	107.1 ± 62.0 (median 95.0)
Per person	48.7 ± 27.1 (median 40.8)
Garden or balcony	909 (95.0%)
Severe physical health problem (diabetes, cancer, etc.)	87 (9.1%)
Mental health problems, lifetime	170 (17.8%)
Current psychiatric treatment	65 (6.8%)
Current psychological/psychotherapeutic treatment	98 (10.2%)
Psychological distress (GSI T-Score ≥ 63)	132(14.4%)
**Loneliness (TILS)**	
Moderate	284 (30.9%)
Severe	208 (22.6%)

One hundred seventy participants indicated to have suffered from mental illness once in their lives. At the time of the survey, 65 (98) individuals had been undergoing psychiatric and/or psychological/psychotherapeutic treatment, respectively.

### COVID-19 Related Variables

Out of 961 study participants 269 had been tested for SARS-CoV-2 (28.3 %) with 18 (1.9%) having had a positive test result. None of the participants was hospitalized due to symptoms associated with COVID-19. With 85.9%, the large majority believed that the containment measures of the COVID-19 pandemic had been adequate and 95.6% indicated to adhere to them. However, 178 individuals (18.5%) felt stressed by the intensified presence of the police.

One hundred ninety-one participants (19.9%) stated that they had consumed alcohol or other substances since the outbreak of the pandemic in order to feel better. Thirteen individuals (1.4%) felt exposed to violence and 66 (6.9%) reported that their propensity to violence had increased during the confinement. Table 2 shows the COVID-19 related variables in detail.

**Table 2:** COVID-19 related variables.

**Variable**	***N* (%)**
**SARS-CoV-2 test**	
Not performed	692 (71.7%)
Negative test result	243 (25.6%)
Positive test result	18 (1.9%)
Result unknown/ not specified	8 (0.8%)
**Severity of COVID-19 Symptoms (*****n*** **=** **18)**	
No symptoms	5 (27.8/0.5%)^a^
Mild symptoms	8 (44.4/0.9%)^a^
Symptoms with fever, treatment at home	5 (27.8/0.5%)^a^
Strong symptoms, treatment in the hospital	0 (0.0/0.0%)^a^
**Do you believe that the measures for the containment of the COVID-19 pandemic are adequate?**	
Yes, entirely	452 (47.0%)
Rather yes	374 (38.9%)
Neither nor	42 (4.4%)
Rather not	66 (6.9%)
Not at all	23 (2.4%)
**Do you adhere to the recommended measures for the containment of the COVID-19 pandemic?**	
Yes, entirely	491 (51.1%)
Rather yes	428 (44.5%)
Neither nor	12 (1.2%)
Rather not	22 (2.3%)
Not at all	3 (0.3%)
Did you consume alcohol or other substances since the outbreak of the COVID-19 pandemic in order to feel better?	191 (19.9%)
Is the intensified presence of the police incriminating for you?	178 (18.5%)
Did/do you feel exposed to violence?	13 (1.4%)
Has your propensity for violence increased?	66 (6.9%)

a *The first percentage refers to the n=18 respondents with positive test results, the second percentage refers to the total sample (N = 961)*.

### Psychological Distress in the Total Sample and in Individual Subgroups

GSI T Scores were available from 914 participants. 14.4% reached scores ≥ 63 and were considered as psychologically distressed. As Table 3 shows, there was a significantly higher risk to suffer from psychological distress among women compared to men and among singles or people in a relationship but not living together compared to people in a relationship living together. Also, low-income participants and homemakers as well as those who were retired, unemployed or working from home during the pandemic had a significantly increased risk of psychological distress. The same was true for participants who had consumed alcohol or other substances since the outbreak of the pandemic in order to feel better.

**Table 3:** Psychological distress in the total sample and in individual subgroups.

**Group/subgroup**	**Percentage with elevated levels of psychological distress^a^**	**Chi-square**	**d.f**.	**Odds ratio**	***p*-value**
Total sample	14.4% (132/914)	–	–	–	–
Gender		5.62	1		0.018
Male	10.3% (29/281)			1.00^b^	
**Female^c^**	**16.3% (103/632)**			**1.69**	
Age		1.51	3		0.680
18–29 years	15.7% (32/204)			1.20	
30–49 years	13.9% (59/425)			1.04	
50–69 years	13.4% (34/254)			1.00^b^	
*≥ 70 years^d^*	*20.7% (6/29)*			*1.69*	
Relationship		22.60	2		<0.001
**Single**	**22.7% (54/238)**			**2.52**	
Partnership, living together	10.3% (59/574)			1.00^b^	
**Partnership, not living together**	**18.6% (19/102)**			**1.99**	
Children		4.68	2		0.096
0	16.3% (98/603)			1.00^b^	
1–2	11.2% (30/269)			0.65	
3–4	9.8% (4/41)			0.56	
Household income		29.22	2		<0.001
** <25,000 per year**	**22.1% (73/330)**			**2.37**	
25,000–50,000 per year	10.7% (37/346)			1.00^b^	
≥50,000 per year	7.1% (15/210)			0.64	
Flat size		0.62	2		0.733
≤ 35 m^2^/person	14.9% (47/316)			1.14	
35.1–50 m^2^/person	13.3% (35/264)			1.00^b^	
>50 m^2^/person	12.8% (36/282)			0.96	
Work situation		51.84	8		<0.001
Full-time or part-time work	10.8% (68/627)			1.00^b^	
Self-employed	11.4% (5/44)			1.06	
Short-time work	14.3% (3/21)			1.38	
From home	**46.2% (6/13)**			**7.09**	
**Unemployed**	**66.7% (6/9)**			**16.54**	
Training/education	16.7% (9/54)			1.65	
**Homemaker**	**31.3% (5/16)**			**3.76**	
**Retired**	**20.0% (18/90)**			**2.06**	
**Others^e^**	**30.8% (12/39)**			**3.65**	
Place of residence		0.57	2		0.753
Urban (Innsbruck)	13.2% (39/295)			1.00	
Rural or small town	14.8% (84/569)			1.13	
Places with high exposition to COVID-19	16.7% (7/42)			1.32	
SARS-CoV-2 test		1.63	2		0.444
Not performed	13.7% (91/661)			1.00^b^	
Test result negative	15.6% (36/231)			1.17	
*Test result positive^d^*	*23.5% (4/17)*			*1.94*	
Consumption of alcohol or other substances since the outbreak of the COVID-19 pandemic in order to feel better		85.99	1		<0.001
No	9.1% (67/735)			1.00^b^	
**Yes**	**36.3% (65/175)**			**5.68**	

a *GSI T-score ≥ 63*.

b *Reference group*.

c *Subgroups shown in bold print had significantly increased levels of psychological distress compared to the reference group*.

d *Subgroups shown in italics had numerically high prevalences of psychological distress (>20%), but did not attain statistical significance (possibly due to a small sample sizes)*.

e *Including sick leave, rehabilitation, maternity leave, among others*.

Belonging to a certain age group, place of residence urban or rural, and having been tested for SARS-CoV-2 test were not associated with higher GSI T scores and similarly, residents of places with high exposition to COVID-19 did not suffer from higher psychological distress than residents of other places in Tyrol.

Analysis of the combined effects of sociodemographics and COVID-19 related variables on psychological distress by logistic regression showed that household income, work situation as well as the consumption of alcohol and other substances remained in the model as significant predictors, whereas the significance of gender and being in a relationship was lost (Table 4).

**Table 4:** Predictors of psychological distress – results of logistic regression.

**Independent variables**	**Beta**	**S.E**.	**Wald Chi-square**	**d.f**.	**Odds ratio**	***p*-value**
**Sociodemographic variables, included as potential confounders**						
Age	−0.007	0.011	0.403	1	0.993	0.526
**Gender**						
Female	0.180	0.263	0.468	1	1.197	0.494
Male (reference)	0	–	–	–	1.000	–
**Significant predictors**						
Household income			13.865	2		0.001
<25,000 per year	1.072	0.333	10.364	1	2.921	0.001
25,000–50,000 per year	0.346	0.252	1.885	1	1.413	0.170
>50,000 per year (reference)	0	–	–		1.000	–
Employment status			27.171	8		0.001
Full-time or part-time work (ref.)	0	–	–	–	1.000	–
Self-employed	−0.128	0.465	0.075	1	0.880	0.784
Short-time work	−0.171	0.692	0.061	1	0.843	0.805
Home office	1.519	0.701	4.691	1	4.567	0.030
Unemployed	2.202	0.754	8.536	1	9.039	0.003
Training/education	0.342	0.535	0.408	1	1.407	0.523
Homemaker	1.457	0.401	13.192	1	4.294	<0.001
Retired	0.870	0.439	3.934	1	2.387	0.047
Others	0.791	0.691	1.312	1	2.206	0.252
Consumption of alcohol or other substances since the outbreak of the pandemic in order to feel better	1.746	0.224	60.881	1	5.732	<0.001

*Not included in the model (p > 0.05): relationship (Wald = 4.373, d.f. = 2, p = 0.112)*.

*S.E., standard error; d.f., degrees of freedom*.

*Model information: Chi-square = 120.2, d.f. = 13, p < 0.001, Nagelkerke R^2^ = 0.228*.

### Severe Loneliness in the Total Sample and in Individual Subgroups

More than a fifth (22.6%) of participants reached a TILS score ≥ 7 and was therefore classified as severely lonely, as shown in Table 5. A higher risk to be affected from severe loneliness was observed in women, singles, people aged 18–49, individuals with low income or living in a small flat, and in people who were unemployed or working from home. In addition, the risk to suffer from loneliness was significantly higher in study participants who had consumed alcohol or other substances since the outbreak of the pandemic in order to feel better.

**Table 5:** Severe loneliness in the total sample and in individual subgroups.

**Group/subgroup**	**Percentage suffering from severe loneliness^a^**	**Chi-square**	**d.f**.	**Odds ratio**	***p*-value**
Total sample	22.6 % (208/919)	–	–	–	–
Gender		22.63	1		<0.001
Male	12.7% (38/283)			1.00^b^	
**Female^c^**	**26.9% (171/635)**			**2.53**	
Age		18.20	3		<0.001
**18**–**29 years**	**30.0% (61/203)**			**2.53**	
**30**–**49 years**	**24.3% (104/428)**			**1.89**	
50–69 years	14.5% (37/256)			1.00^b^	
≥70 years	13.8% (4/29)			0.96	
Relationship		24.86	2		<0.001
**Single**	**33.6% (80/238)**			**2.37**	
Partnership, living together	17.6% (102/578)			1.00^b^	
Partnership, not living together	24.5% (25/102)			1.52	
Children		3.01	2		0.222
0	22.6% (137/606)			1.00^b^	
1–2	24.4% (66/271)			1.09	
≥3	12.2% (5/41)			0.49	
Household income		6.21	2		0.045
**<25,000 per year**	**27.3% (90/330)**			**1.52**	
25,000–50,000 per year	19.8% (69/348)			1.00^b^	
≥50,000 per year	20.4% (43/211)			1.04	
Flat size		6.09	2		0.048
**≤35 m**^**2**^**/person**	**24.2% (77/318)**			**1.56**	
**35.1**–**50 m**^**2**^**/person**	**24.5% (65/265)**			**1.58**	
>50 m^2^/person	17.0% (48/283)			1.00^b^	
Work situation		27.59	8		<0.001
Full-time or part-time work	21.4% (135/631)			1.00^b^	
Self-employed	11.4% (5/44)			0.52	
Short-time work	19.0% (4/21)			0.87	
**From home**	**46.2% (6/13)**			**3.15**	
**Unemployed**	**77.8% (7/9)**			**12.86**	
Training/education	25.9% (14/54)			1.28	
Homemaker	18.8% (3/16)			0.85	
Retired	22.2% (20/90)			1.05	
**Others^d^**	**35.0% (14/40)**			**3.65**	
Place of residence		1.93	2		0.382
Urban (Innsbruck)	25.0% (74/296)			1.00	
Rural or small town	22.0% (126/573)			0.92	
Places with high exposition to COVID-19	16.7% (7/42)			0.60	
SARS-CoV-2 test		0.01	2		0.995
Not performed	22.7% (151/666)			1.00^b^	
Test result negative	22.8% (53/232)			1.01	
Test result positive	23.5% (4/17)			1.04	
Consumption of alcohol or other substances since the outbreak of the COVID-19 pandemic in order to feel better		36.13	1		<0.001
No	18.5% (137/7395)			1.00^b^	
**Yes**	**39.4% (71/180)**			**2.86**	

a *TILS total score ≥ 7*.

b *Reference group*.

c *Subgroups shown in bold print had significantly increased levels of loneliness compared to the reference group*.

d *Including sick leave, rehabilitation, maternity leave, among others*.

When analyzing the combined effects of sociodemographic and COVID-19 related variables on loneliness, the variables age group, gender, being in a relationship, work situation, flat size, and consumption of alcohol or other substances were retained as significant predictors, while the significance of household income was lost (Table 6).

**Table 6:** Predictors of severe loneliness – results of logistic regression.

**Independent variables**	**Beta**	**s.e**.	**Wald Chi-square**	**d.f**.	**Odds ratio**	***p*-value**
Age	0.023	0.009	6.158	1	0.978	0.013
**Gender**						
Female	0.930	0.233	15.870	1	2.535	<0.001
Male (reference)	0	–	–	–	1.000	–
Relationship			14.894	2		0.001
Partnership, living together (reference)	0	–	–	–	1.000	–
Partnership, not living together	0.387	0.317	1.490	1	1.473	0.222
Single	0.848	0.220	14.875	1	2.335	<0.001
Flat size			8.867	2		0.012
<35 m^2^/person	0.656	0.253	6.736	1	1.928	0.009
35–50 m^2^/person	0.677	0.245	7.647	1	1.968	0.006
>50 m^2^/person (reference)	0	–	–	–	1.000	–
Employment status			15.659	8		0.048
Full-time or part-time work (reference)						
Self-employed	−0.726	0.449	2.610	1	0.484	0.106
Short-time work	−0.297	0.594	0.250	1	0.743	0.617
Home office	0.693	0.691	1.007	1	2.000	0.316
Unemployed	2.502	1.128	4.916	1	12.202	0.027
Training/education	−0.555	0.579	0.919	1	0.574	0.338
Homemaker	0.697	0.377	3.426	1	2.008	0.064
Retired	0.612	0.393	2.424	1	1.845	0.120
Others	0.024	0.698	0.001	1	1.025	0.972
Consumption of alcohol or other substances since the outbreak of the pandemic in order to feel better	0.984	0.209	22.241	1	2.675	<0.001

*Not included in the model (p > 0.05): Income (Wald Chi-square = 4.239, d.f. = 2, p = 0.120)*.

*S.E., standard error; d.f., degrees of freedom*.

*Model information: Chi-square = 103.0, d.f. = 15, p < 0.001, Nagelkerke R^2^ = 0.178*.

### Boredom in the Total Sample and in Individual Subgroups

As shown in Table 7, the study population reached a mean of 25.9 ± 11.0 points in the MSBS-SF. With a mean of 33.2 ± 9.2 points unemployed people suffered the most from boredom, followed by people who had consumed alcohol or other substances since the outbreak of the pandemic in order to feel better (32.8 ± 11.0) and people with a positive SARS-CoV-2 test result (32.4 ± 12.9). Also, women, people aged 18–49, singles, people in a relationship but not living together, childless and low-income individuals, those working from home or being in education as well as those living in cities were more burdened from boredom than the remaining sample.

**Table 7:** Boredom in the total sample and in individual subgroups.

**Group/subgroup**	**Mean ± SD**	**Test statistic**	**d.f**.	**Effect size, d**	***p*-value**
Total sample	25.9 ± 11.0	–	–	–	–
Gender		*t* = 2.99	1		0.003
Male	24.3 ± 10.5			0.00^a^	
**Female^b^**	**26.6** **±** **11.3**			**0.21**	
Age		*F* = 15.71	3		<0.001
**18**–**29 years**	**30.0** **±** **11.8**			**0.61**	
**30**–**49 years**	**25.6** **±** **10.7**			**0.23**	
50–69 years	23.3 ± 9.8			0.00^a^	
≥70 years	24.5 ± 10.6			0.12	
Relationship		*F* = 16.90	2		<0.001
**Single**	**28.2** **±** **11.5**			**0.35**	
Partnership, living together	24.4 ± 10.4			0.00^a^	
**Partnership, not living together**	**29.4** **±** **11.4**			**0.45**	
Children		*F* = 10.20	2		<0.001
**0**	**27.0** **±** **11.5**			**0.26**	
1–2	24.1 ± 10.5			0.00^a^	
≥3	21.6 ± 8.9			−0.23	
Household income		*F* = 10.63	2		<0.001
**<25,000 per year**	**28.5** **±** **11.3**			**0.27**	
25,000–50,000 per year	25.5 ± 10.7			0.00^a^	
≥50,000 per year	23.8 ± 10.7			−0.16	
Flat size		*F* = 1.47	2		0.231
≤ 35 m^2^/person	26.5 ± 11.5			0.14	
35.1–50 m^2^/person	25.6 ± 10.3			0.05	
>50 m^2^/person	25.0 ± 10.9			0.00^a^	
Work situation		*F* = 2.83	8		0.004
Full-time or part-time work	25.1 ± 10.7			0.00^a^	
Self-employed	25.5 ± 8.5			0.04	
Short-time work	28.4 ± 12.2			0.30	
**From home**	**32.1** **±** **13.2**			**0.63**	
**Unemployed**	**33.2** **±** **9.2**			**0.73**	
**Training/education**	**29.7** **±** **13.2**			**0.42**	
Homemaker	27.1 ± 11.0			0.18	
Retired	26.7 ± 10.4			0.15	
Others^c^	28.8 ± 12.5			0.34	
Place of residence		*F* = 3.23	2		0.040
**Urban (Innsbruck)**	**27.2** **±** **10.7**			**0.19**	
Rural or small town	25.3 ± 11.1			0.00^a^	
Places with high exposition to COVID-19	26.7 ± 10.8			0.13	
SARS-CoV-2 test		*F* = 3.15	2		0.043
Not performed	25.9 ± 11.1			0.00^a^	
Test result negative	25.8 ± 10.3			0.01	
**Test result positive**	**32.4** **±** **12.9**			**0.59**	
Consumption of alcohol or other substances since the outbreak of the COVID-19 pandemic in order to feel better		*t* = 9.88	1		<0.001
No	24.3 ± 10.4			0.00^a^	
**Yes**	**32.8** ± 11.0			**0.77**	

a *Reference group*.

b *Subgroups shown in bold print had significantly increased levels of boredom compared to the reference group*.

c *Including sick leave, rehabilitation, maternity leave, among others*.

When analyzing the joint effects of sociodemographic and COVID-19 related variables on boredom by multiple linear regression, the variables age group, being in a relationship but not living together, work situation and consumption of alcohol or other substances remained in the model as significant predictors, whereas gender, household income, place of residence (urban vs. rural) and SARS-CoV-2 test result were no longer significant (Table 8).

**Table 8:** Predictors of boredom – results of multiple linear regression.

**Independent variables**	**Beta^a^**	**S.E**.	**F**	**t**	**d.f**.	***p*-value**
**Socio-demographic variables**						
Age group			12.28		3	<0.001
18–29	7.825	2.390		3.27	1	0.001
30–49	3.611	2.285		1.58	1	0.114
50–69	1.298	2.143		0.6	1	0.545
70+	0	–	–	–	–	–
Gender			1.80		1	0.180
Female						0.180
Male (reference)	0	–	–	–	–	–
**Significant predictors**						
Relationship			5.69		2	0.004
Partnership, living together (reference)	0	–	–	–	–	–
Partnership, not living together	2.162	0.804		2.69	1	0.007
Single	2.872	1.099		2.61	1	0.009
Employment status			2.32		8	0.018
Full-time or part-time work (reference)	0	–	–	–	–	–
Self-employed	−0.407	1.564		−0.26		0.795
Short-time work	2.361	2.190		1.08		0.281
Home office	5.679	2.850		1.99		0.047
Unemployed	4.344	3.412		1.27		0.203
Training/education	2.199	1.593		1.38		0.168
Homemaker	3.615	1.656		2.18		0.029
Retired	4.054	1.447		2.80		0.005
Consumption of alcohol or other substances since the outbreak of the pandemic in order to feel better	7.801	0.848		9.20	1	<0.001

a *Unstandardized regression coefficient*.

*s.e., standard error; d.f., degrees of freedom*.

*Overall model information: adjusted R^2^ = 0.156, F= 12.52, d.f. = 15, p < 0.001*.

### Testing Common Method Bias

An EFA based on the dependent variables psychological distress, loneliness and boredom as well as COVID-19 related independent variables (COVID-19 related alcohol consumption, SARS-Cov2 test, opinions regarding violence, presence of police etc., see Table 2) gave rise to a proportion of 27.4% of the total variance explained by the first factor. When reducing the set of COVID-19 related independent variables to those used in Tables 3–8 (COVID-19 related alcohol consumption and SARS-Cov2 test) the proportion of variance explained by the first factor rose to 42.4%, but still remained below 50%. Hence, there was no indication of appreciable common method bias.

## Discussion

Out of 961 study participants from the general population of Tyrol 14.4% reached a GSI T score ≥ 63 and could therefore be considered as severely distressed, which corroborates the findings of a recent study from the United States (46, 47). 22.6% suffered from severe loneliness (TILS score ≥ 7), and boredom levels lay by a mean of 25.9 ± 11.0 points in the MSBS-SF (range: 7–56). Importantly, women, singles, low-income people as well as those who were unemployed were significantly more often affected by all of the selected outcomes compared to the remaining sample and they had significantly more frequently consumed alcohol or other substances since the outbreak of the pandemic in order to feel better. Our findings on psychological distress related to COVID-19 largely support those of previous investigations from different countries (30, 34, 35, 48). Rossi et al. (34), for example, investigated a large sample from the general population of Italy and found a higher risk for different mental health outcomes including perceived stress among women and those experiencing working, financial, relationship, or housing problems. Similarly, the majority of seriously distressed people from the United States reported that pandemic-related disruptions of education, employment, and finances negatively affected their mental health (46), and Pieh et al. (35), who had investigated a representative sample of the Austrian general population found highest mental health problems among women as well as unemployed and low-income people, i.e., among individuals who are generally known to be at increased risk of impaired mental health (49, 50). In addition, they found younger study participants to be most burdened, which is again in agreement with the above mentioned survey from the United States (46) and is also reflected in our finding of loneliness and boredom being most prevalent amongst those from 18 up to the age of 29 (30%, each), followed by the age group from 30 to 49 (24.3 and 25.6%, respectively). However, belonging to a certain age group was not associated with a higher degree of psychological distress as assessed by the BSCL in our sample and accordingly, loneliness and boredom may represent less salient stressors compared to e.g., pandemic-associated working or financial problems.

Nonetheless, loneliness has been one of the most frequently identified personal stress factors during this pandemic (28). Of note, more than one fifth of our sample reported to suffer from severe loneliness, whereas in an earlier population study from Denmark merely 4.6% scored above the same cut-off of the TILS (42). A higher risk to be affected from severe loneliness was found among singles and people who were unemployed or working from home as well as among those with low income or living in a small flat, which is in line with the findings of earlier studies (51). Furthermore, as mentioned above, loneliness was especially prevalent in younger study participants, which also corroborates the findings of other surveys from different countries, e.g., the United States (52), the United Kingdom (53), and Norway (51). Young and middle-aged adults have previously been shown to need more social contacts (51) and to be motivated to build and expand their social network outside their family of origin (54), which is why they may be particularly affected by loneliness in the context of pandemic-related restrictions.

During the SARS outbreak in 2003, feeling bored has been the biggest challenge in complying with quarantine regulations (12). In the context of the COVID-19 pandemic, boredom has been the most common reported feeling in China, followed by anxiety and worry, and the degree of boredom correlated with the occurrence of depression, anxiety, and stress (13). On the other hand, boredom can be a key emotion enabling people to change their behavior and thus reach a more satisfying situation (55). During a pandemic, however, the state of boredom may be associated with negative aspects. People tend to have more time available but cannot use it as desired because of isolation and restrictions, which may subsequently lead to depression (33). The possibility to counteract boredom by changing behavior is often prevented by social isolation (56). Accordingly, our finding of boredom being most prevalent among those who were unemployed, single or in a relationship but not living together, childless, or working from home is not surprising. People living in more rural areas may probably have had more opportunities to escape the quarantine situation and to positively change their behavior, which is why urban residents may have been more burdened by boredom among our sample.

It has previously been suggested that due to a lack of external stimulation, bored individuals may develop a tendency toward anger, outbursts of rage, aggression, and deficits in anger control (57). This could not be confirmed in our study with merely 66 out of 961 respondents stating that their propensity to violence had increased during the pandemic. However, when interpreting the data presented in this paper, one has to consider that they have been collected in the early stages of the pandemic and it remains to be seen whether this will change in the long-term.

Previous studies have shown that loneliness, social isolation and/or a change in employment can trigger substance use in susceptible individuals, which can manifest itself as a worsening in existing addictions or as a relapse after abstinence (22, 58). It is alarming that one fifth of our sample stated that they had consumed alcohol or other substances in order to feel better and studies from Belgium and Australia found even higher rates of substance use to cope with mental stress during the COVID-19 pandemic (59). Notably, study participants who had consumed alcohol or other substances since the outbreak of the pandemic were more frequently affected by psychological distress (36.3%; OR = 5.68), loneliness (39.4%, OR = 2.86), and boredom (32.8%, d = 0.77). Longitudinal data are needed to investigate whether these outcomes persist after the COVID-19 pandemic and whether this may lead to an increase of long-lasting mental health problems including drug use in the general population. At the same time, it will be critical to expand mental health services to serve those most at risk and identify options of maintaining social contacts and remaining active despite pandemic-related restrictions.

Notwithstanding the implications of our findings, there are a number of limitations that should be considered. First, we conducted an online survey and people who were not reached by advertising as well as those who have problems with internet usage could not participate in the survey. However, we tried to reach a heterogeneous group of the Tyrolean population from all socio-economic backgrounds. This was done through advertisements in different local newspapers and various social media. In addition, posters and flyers were used to draw attention to the study. Of course, a distortion of the results cannot be ruled out, as people with a higher burden may have been more likely to participate in the study. Moreover, merely 1.3% of study participants stated to work from home, whereas a recent study found that at least half of the Austrian workforce worked from home during the pandemic (60). Accordingly, a **sample bias** has to be taken into account, which limits the generalizability of the obtained results. Secondly, the information obtained was obviously self-reported, which can result in social desirability bias. An additional limitation is the lack of knowledge about levels of psychological distress, loneliness, and boredom before the COVID-19 outbreak and causal relationships can therefore not be deduced from these data. Moreover, the heterogeneity of study participants was relatively high in terms of age, living conditions, or socioeconomic status. Therefore, we cannot exclude the possibility that subgroups differ in terms of the parameters surveyed. However, due to the longitudinal design of this study we will be able to collect follow-up data to explore how the investigated issues change in the course of the pandemic.

Notwithstanding these limitations, our findings reemphasize the relevance of promoting mental health during the COVID-19 pandemic.

## Conclusion

Our results suggest that women, singles, low-income people as well as those who are unemployed may particularly be affected by psychological distress, loneliness, and boredom during the COVID-19 pandemic and that they are exposed to an increased risk of substance use in order to feel better. In addition, young and middle-aged adults may specifically be burdened by loneliness and boredom. In order to prevent mental health problems it will be critical to identify options of maintaining social contacts and remaining active despite pandemic-related restrictions.

## Data Availability Statement

The datasets presented in this article are not readily available because its proprietary nature or ethical concerns. Requests to access the datasets should be directed to Franziska Tutzer, franziska.tutzer@i-med.ac.at.

## Ethics Statement

The studies involving human participants were reviewed and approved by ethics committee of the Medical University Innsbruck. The patients/participants provided their written informed consent to participate in this study.

## Author Contributions

AH, BF-A, SP, BH, and BP designed the study and wrote the protocol. Recruitment was performed by FT and AC. GK undertook statistical analysis. FT wrote the first draft of the manuscript. All authors contributed to and have approved the final manuscript.

## Conflict of Interest

BH owns part of the IPRs of the CHES software tool. The remaining authors declare that the research was conducted in the absence of any commercial or financial relationships that could be construed as a potential conflict of interest.
